# Prevalence of Attention Deficit Hyperactivity Disorder in Detention Settings: A Systematic Review and Meta-Analysis

**DOI:** 10.3389/fpsyt.2018.00331

**Published:** 2018-08-02

**Authors:** Stéphanie Baggio, Ana Fructuoso, Marta Guimaraes, Eveline Fois, Diane Golay, Patrick Heller, Nader Perroud, Candy Aubry, Susan Young, Didier Delessert, Laurent Gétaz, Nguyen T. Tran, Hans Wolff

**Affiliations:** ^1^Division of Prison Health, Geneva University Hospitals and University of Geneva, Geneva, Switzerland; ^2^Life Course and Social Inequality Research Centre, University of Lausanne, Lausanne, Switzerland; ^3^Division of Psychiatric Specialties, Department of Mental Health and Psychiatry, University Hospitals of Geneva, Geneva, Switzerland; ^4^Foundation TDAH-Insight and Association TDAH-ImPulse, Geneva, Switzerland; ^5^Division of Brain Sciences, Department of Medicine, Centre for Mental Health, Imperial College London, London, United Kingdom; ^6^Prison Health Care, Valais Hospital, Sion, Switzerland; ^7^Faculty of Health, Australian Centre for Public and Population Health Research, University of Technology, Sydney, NSW, Australia

**Keywords:** ADHD, incarceration, offender, prevalence, prison

## Abstract

**Background:** Previous studies have reported a high prevalence of attention deficit hyperactivity disorder (ADHD) among people living in detention (PLD) corresponding to a five- to ten-fold increase compared to the general population. Our main study objective was to provide an updated ADHD prevalence rate for PLD, including PLD in psychiatric units. Sub-objectives included (i) comparing different ways of assessing ADHD, including DSM-5 criteria and (ii) identifying which types of PLD are more likely to have ADHD.

**Methods:** We conducted a systematic review and meta-analysis following the PRISMA guidelines and the MOOSE checklist. PubMed/Medline, PsycINFO, and Web of Sciences were searched combining “ADHD” and “prison” keywords and synonyms for articles published between January 1, 1966 and January 2, 2018. Potential sources of variation to the meta-analytic ADHD prevalence rate were investigated using meta-regressions and subgroups analyses.

**Results:** The meta-analysis pooled 102 original studies including 69,997 participants. The adult ADHD prevalence rate was 26.2% (95% confidence interval: 22.7–29.6). Retrospective assessments of ADHD in childhood were associated with an increased prevalence estimate (41.1, 95% confidence interval: 34.9–47.2, *p* < 0.001). There was no significant difference in the prevalence estimate between screenings and clinical interviews in adulthood. Only three studies used the DSM-5 definition of ADHD and results were non-significantly different with other DSM versions. We found no difference according to participants' characteristics.

**Conclusion:** Our results confirmed the high prevalence rate of ADHD among PLD, corresponding to a five-fold increase compared to the general population. In light of such high ADHD prevalence, our results reinforce the importance of addressing this critical public health issue by (i) systematically offering ADHD screening and diagnosis to all individuals entering detention, and (ii) delivering treatment, monitoring, and care for ADHD during and after detention. These strategies may help reduce recidivism and reincarceration, as well as violence in detention settings, in addition to improving the health and wellbeing of people living in detention. Additionally, our study suggests that using screening scales may be a reliable way of assessing ADHD, although caution is needed because a complete evaluation by an experienced clinician is required to provide a formal diagnosis.

## Introduction

### ADHD in the general population

Attention deficit hyperactivity disorder (ADHD) is a disorder characterized by difficulties paying attention, poor impulse control, and hyperactive behaviors. ADHD starts in early childhood and persists in adulthood in 40–60% of cases (1). There is growing evidence that adult ADHD is a major health concern (2). It is associated with at-risk behaviors and comorbid psychiatric disorders (3) and affects several areas of life, such as psychosocial functioning, school, work, and health care access and health care use (4).

### ADHD in incarcerated population

ADHD is associated with an increased risk of having judicial contact at a younger age, including rule-breaking behaviors, delinquency, criminality, and recidivism (5–7). ADHD seems to be significantly more prevalent in incarcerated populations in comparison with the general population and it has been extensively studied in detention settings over the two last decades (5). Compared with other offenders, incarcerated individuals with ADHD are more likely to engage in misconduct in prison, for example, be verbally and physically aggressive (8, 9), have higher rates of recidivism (10), and have unsuccessful experiences with the criminal justice system as well as with probation (11). Therefore, ADHD seems to be a critical factor of the criminal career (7), but further investigations are needed to understand how ADHD is associated with involvement in the legal system.

To date, the only meta-analysis reporting ADHD prevalence in incarcerated populations included studies published until 2012. The study identified a five- to ten-fold increase in prevalence of ADHD compared to the general population (5): 25.5% compared to 5% in the general population (12–14). Since 2012, several studies have investigated the prevalence rates of ADHD in people living in detention (PLD) worldwide. Additionally, this meta-analysis did not include PLD detained in psychiatric units and therefore PLD with formal diagnostic of comorbid psychiatric disorders were likely to be excluded. A more complete picture of ADHD in prison setting is therefore needed.

### Measures of ADHD

ADHD was introduced for the first time in the second version of the Diagnostic and Statistical Manual of Mental Disorders (DSM-II), as “kyperkinetic disorder of childhood” (15). It emphasized on hyperactivity as a cardinal feature of the disorder. In the subsequent version of the DSM (DSM-III), the disorder was labeled “Attention deficit disorder with or without hyperactivity” (16). It emphasized on the attentional aspects of the disorder, being considered as a tri-dimensional disorder. However, subtypes were not considered. The main changes introduced in the DSM-IV (17) were to label the disorder “ADHD” and to define three subtypes (inattentive, hyperactive, and combined). Then, two major changes in the diagnostic criteria for adult ADHD were introduced in the fifth version of the DSM (DSM-5), which may affect the prevalence rate of ADHD (18). In the DSM-5, there are a reduced number of symptoms for the diagnosis in adults (five instead of six) and a later age of onset (twelve instead of six) needed to diagnose ADHD. These changes to the DSM-IV aim to address the restrictive diagnostic thresholds (19) and the late onset of some symptoms that may occur in adulthood (20). Recent studies concluded that the switch from DSM-IV to DSM-5 diagnostic threshold resulted in a modest increase and less biased ADHD prevalence rate (4, 21).

To date, no systematic review and meta-analysis has provided an overview of how DSM-5 criteria may have affected the prevalence rate of ADHD, especially among PLD. Furthermore, a recent article questioned the reliability of ADHD prevalence rate among PLD, as some major methodological shortcomings, such as self-reported assessments or non-representative sampling, may have resulted in high prevalence rates (22). Evidence regarding the quality of ADHD studies in prison was therefore needed.

### Objective of the study

This meta-analysis aimed to provide an updated estimate the prevalence rate of ADHD in PLD over the past three decades, including articles published since 2012. Sub-objectives included (i) comparing different ways of assessing ADHD and in particular investigating whether the DSM-5 resulted in an increased prevalence rate of ADHD, and (ii) identifying which characteristics of PLD were more likely to be associated with ADHD (e.g., socio-demographics).

## Methods

The systematic review and meta-analysis adhered to the Preferred Reporting Items for Systematic Reviews and Meta-Analyses (PRISMA) guidelines (23) and the Meta-analysis of Observational Studies in Epidemiology (MOOSE) checklist (24). The protocol for this review was previously registered on Prospero (CRD42017075510).

### Eligibility criteria

All studies investigating ADHD in PLD were eligible for this systematic review. In addition, articles were eligible if they (i) reported an empirical study, (ii) were written in English, and (iii) were published in a peer-reviewed journal.

### Search strategy

We searched Pubmed/Medline, PsycINFO, and Web of Sciences from their inception date until January 2, 2018. We used the terms “attention deficit hyperactivity disorder” or “ADHD” and “prison” or “prisoner” or “inmate” or “detaine^*^” or “custod^*^” or “detention” or “crim^*^” or “offend^*^” or “correctional” or “forensic” or “penal institution.” Published meta-analyses on the subject identified in the search were hand-searched for other relevant studies using their reference lists and studies quoting them. These meta-analyses were excluded from the calculation of prevalence estimates. Reference lists of retrieved studies were also hand-searched.

### Study selection

After article duplicates were removed, a first round of selection was performed to exclude studies meeting exclusion criteria defined in the subsection Eligibility Criteria by screening titles and abstracts, and, if necessary, the whole article. A second round of selection was performed by reviewing the full text of articles. We excluded articles reporting on the same dataset, articles focusing only on participants with ADHD (100% prevalence rate), articles reporting no prevalence rate (after unsuccessful correspondence with the corresponding author), articles with mixed prevalence rates for males and females, because gender is known as an important predictor of ADHD (25) (for which the corresponding author did not provide an answer regarding separate prevalence rates), or if we were unable to access the article.

Two rounds of reviewers (SB and AF/DG/MG) independently screened all the abstracts in the first selection round. In case of disagreement, consensus was achieved by discussion, and, if required, by a third-party arbitration (HW).

### Data extraction

Characteristics of studies included in the meta-analysis were extracted independently by two rounds of reviewers (SB and EV/MG/NTT) using an electronic data abstraction form on Excel. The form included the following study characteristics: (1) year of publication (we used this information instead of year of data collection because the latter was missing in 43.1% of the studies); (2) geographic location; (3) sample size; (4) study population (adults vs. youths); (5) type of detention setting (prison, youth detention centers, or psychiatric unit); (6) gender; (7) mean age; (8) presence of psychiatric disorders in the sample (sample of psychiatric participants vs. “ordinary” participants); (9) type of offenders (serious vs. non-serious offenders; with “serious offending” defined in the corresponding article as: violent or high-risk PLD, rapists, maximum-security PLD, long-term sentences); (10) diagnostic tools (self-reported screening for ADHD in adulthood/adolescence, self-reported screening for ADHD in childhood, or clinical interview), (11) criteria used for diagnosis (DSM-III, DSM-IV, or DSM-5); and (12) ADHD prevalence rate. Mean age was included for descriptive purposes. Studies involving both gender and/or using different diagnostic criteria (e.g., a self-reported assessment and a clinical interview) were recorded as separate observations.

We contacted the authors of 67 articles regarding missing information. Five authors answered but were unable to provide gender-disaggregated prevalence rates, 35 provided missing information, and 27 did not answer. Studies with missing information on other variables than prevalence rates were kept for descriptive purposes and to estimate the meta-analytic prevalence rate of ADHD—this was not an exclusion criterion. Listwise deletion was used for other analyses.

### Risk of bias assessment

Articles included in the systematic review and meta-analysis were assessed for the risk of bias using an adaptation of the Quality in Prognosis Studies including the following relevant items (26): (1) sample selection, (2) study participation, (3) outcome measurement, and (4) presence of exclusion criteria. Each study was rated as low, moderate, and high quality by two rounds of reviewers (SB and EF/MG/TNT) (see Appendix 1 in Supplementary Materials).

### Statistical analyses

We first undertook a descriptive analysis of the studies. We then estimated the meta-analytic prevalence of ADHD. We provided separate prevalence estimates for studies with an adolescent/adult measure (screenings and clinical interviews) and childhood measure. We also computed the prevalence estimate for studies using clinical interviews, the most reliable and valid way to assess ADHD. Indeed, screening tests are not diagnostic tests (established using clinical interviews). They are designed to detect people at risk for the corresponding disease. Diagnostic tests establish the presence of the disease and are used to determine the need for treatment (27, 28). Finally, we tested potential influences of study characteristics. Covariates were first tested using univariate meta-regressions, and then simultaneously in a multivariate meta-regression for all studies and for studies using clinical interviews. In multivariate analyses, only factors with a sufficient number of observations were included. As prison type was redundant with study population and presence of psychiatric disorders, we excluded it from the analyses. We used random-effects model with restricted maximum-likelihood estimator (29) and the Knap and Hartung method (30). We reported “variance accounted for” (VAF) using a pseudo-R^2^. VAF is an indicator of effect size and corresponds to the percentage of the heterogeneity in the prevalence that is accounted for in each model. Analyses were performed using R 3.4.3 and the package “metafor” version 2.0.0.

## Results

### Study selection

**Figure 1:**
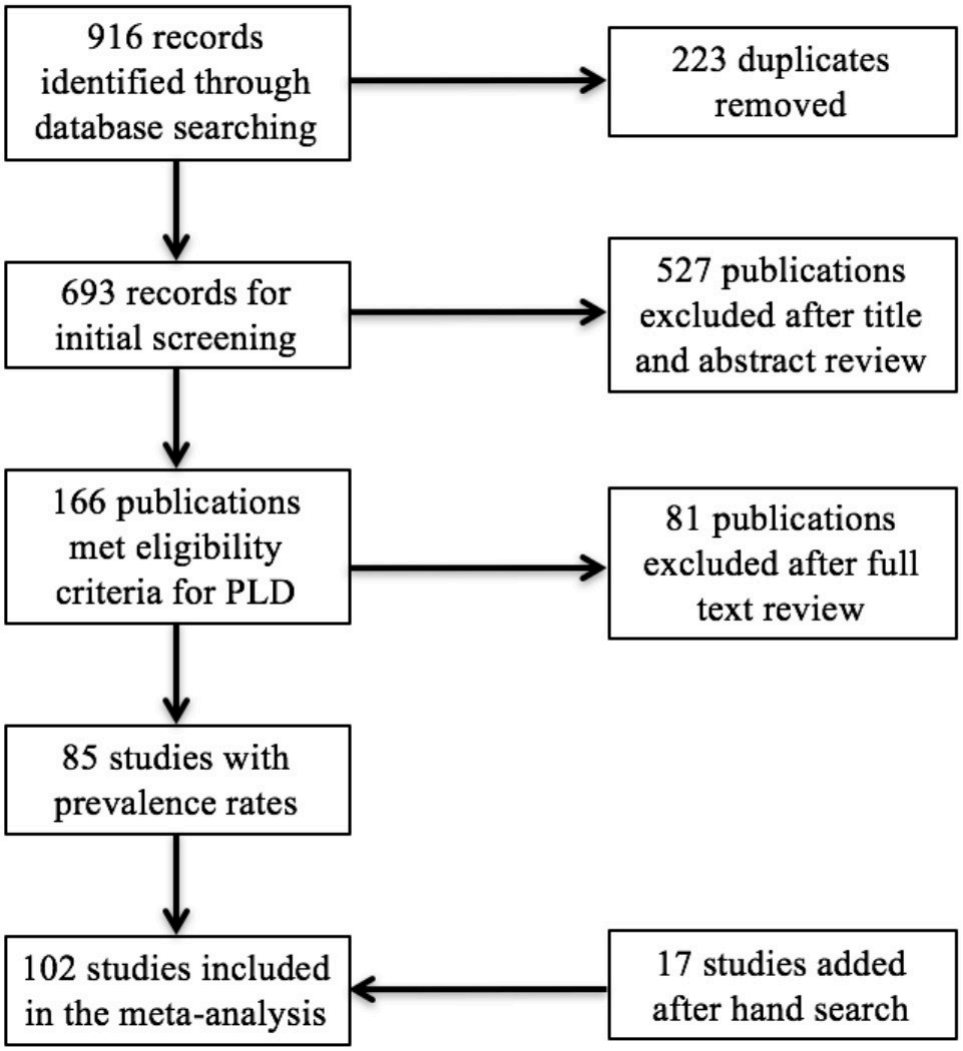
Flow diagram of study screening and inclusion process. PLD, people living in detention.

We identified 916 records on PubMed/Medline, psycINFO, and Web of Sciences. After removal of 223 duplicates, 693 publications remained. We excluded 527 publications after a first screening because they did not focus on ADHD in PLD or did not report empirical findings. After further review of the remaining 166 publications, 81 were excluded: 23 articles did not report ADHD prevalence rates or gender-disaggregated prevalence rates, 8 used samples composed of participants with 100% ADHD, 47 relied on data already used in other articles, and 3 because we had no access to the full article. The manual search of published meta-analyses led to the identification of 17 other studies. A total of 102 publications were included in the meta-analysis (Figure 1). It led to 142 samples: 67 studies with a single sample, 26 studies with both genders, 9 studies with two assessment tools, one study with both genders and two diagnostics, and one study with three diagnostics (one childhood screening, one adulthood screening, and a clinical interview). Data are reported in the Appendix 3. References for all studies are reported in the Appendix 2.

### Studies' characteristics

The meta-analysis pooled 102 original studies (142 samples), including 69,997 participants (males: 89.0%; females: 11.0%; adults: 27.5%, mean age = 32.7, range 24.8–44.9; youths: 72.5%, mean age = 16.4, range 14.0–20.0). A total of 64.7% of the studies were published in the 2008–2017 period, whereas 29.4% were published between 1998 and 2007, and 4.9% between 1988 and 1997, plus one publication in 1985 (1.0%). The 25, 50, and 75th percentiles of year distribution corresponded respectively to years 2004, 2010, and 2014. Data came from 28 countries distributed as follows: Europe (49.0%, *n* = 67), North America (35.3%, *n* = 53), Asia (6.9%, *n* = 8), Australia (4.9%, *n* = 9), and South America (3.9%, *n* = 5). The information on the number of studies included for each region and other characteristics are reported in the first column of in Table 1. Most studies used a clinical diagnosis (58.5%, *n* = 83), while 21.1% (*n* = 30) used self-reported screenings of childhood ADHD and 20.4% (*n* = 29) self-reported screenings of adolescent/adult ADHD. A total of 16.2% (*n* = 23; total number of participants = 2,321, not shown in Table 1) of the studies focused on samples of participants with a psychiatric diagnosis other than ADHD (for example, participants with conduct or personality disorders, schizophrenia, or referred for psychiatric assessment), and 15.5% on serious offenders (*n* = 22; total number of participants = 15,360, not shown in Table 1). Overall, the quality of the studies was high. There was 23.2% (*n* = 33) of studies with a “weak” quality: in total, 13.4% had a response rate ≤60% or a convenient sample, 24.7% excluded non-native speakers, and 16.2% excluded PLD with psychiatric or somatic disorders (e.g., psychotic symptoms, presence of severe mental disorder, or physical illness, but of course participants with ADHD symptoms were not excluded) (not shown in Table 1).

**Table 1:** Univariate and multivariate meta-regressions for all study samples (*n* = 142).

	**No. of study samples**	**Univariate models**			**Multivariate model**		
		**Estimate**	***p*-value**	**VAF%**	**Estimate**	***p*-value**	**VAF5**
Intercept	−	–	–	–	**0.23**	**<0.001**	**7.8**
**Region**							
North America (reference)	53	**0.29**	**<0.001**	1.5	–	–	
Asia	8	0.07	0.331		–	–	
Australia	9	−0.07	0.307		–	–	
Europe	67	0.03	0.453		–	–	
South America	5	−0.14	0.113		–	–	
**Gender**							
Male (reference)	104	**0.30**	**<0.001**	0.0	–	–	
Female	38	−0.01	0.768		−0.01	0.901	
**Study population**							
Adults (reference)	77	**0.31**	**<0.001**	0.0	–	–	
Youths	65	−0.01	0.694		0.03	0.340	
**Psychiatric diagnosis**							
No (reference)	119	**0.29**	**<0.001**	0.0	–	–	
Yes	23	0.04	0.340		0.05	0.228	
**Serious offenders**							
No (reference)	120	**0.30**	**<0.001**	0.0	–	–	
Yes	22	−0.01	0.803		−0.01	0.778	
**Diagnostic**							
Interview (reference)	83	**0.28**	**<0.001**	**10.3**	–	–	
Current screening	29	−0.03	0.574		−0.01	0.924	
Retrospective screening	30	**0.15**	**<0.001**		**0.16**	**0.003**	
**DSM Version**							
DSM-IV (reference)	112	**0.29**	**<0.001**	0.0	–	–	
DSM-III	18	0.02	0.618		–	–	
DSM-5	5	0.08	0.484		–	–	
**Quality**							
Strong (reference)	57	**0.27**	**<0.001**	0.5	–	–	
Moderate	52	0.04	0.328		0.01	0.950	
Weak	33	0.06	0.138		0.02	0.662	

*VAF, Variance accounted for (pseudo-R^2^)*.

*Significant and marginally significant results are in bold*.

### Overall prevalence rate of ADHD

**Figure 2:**
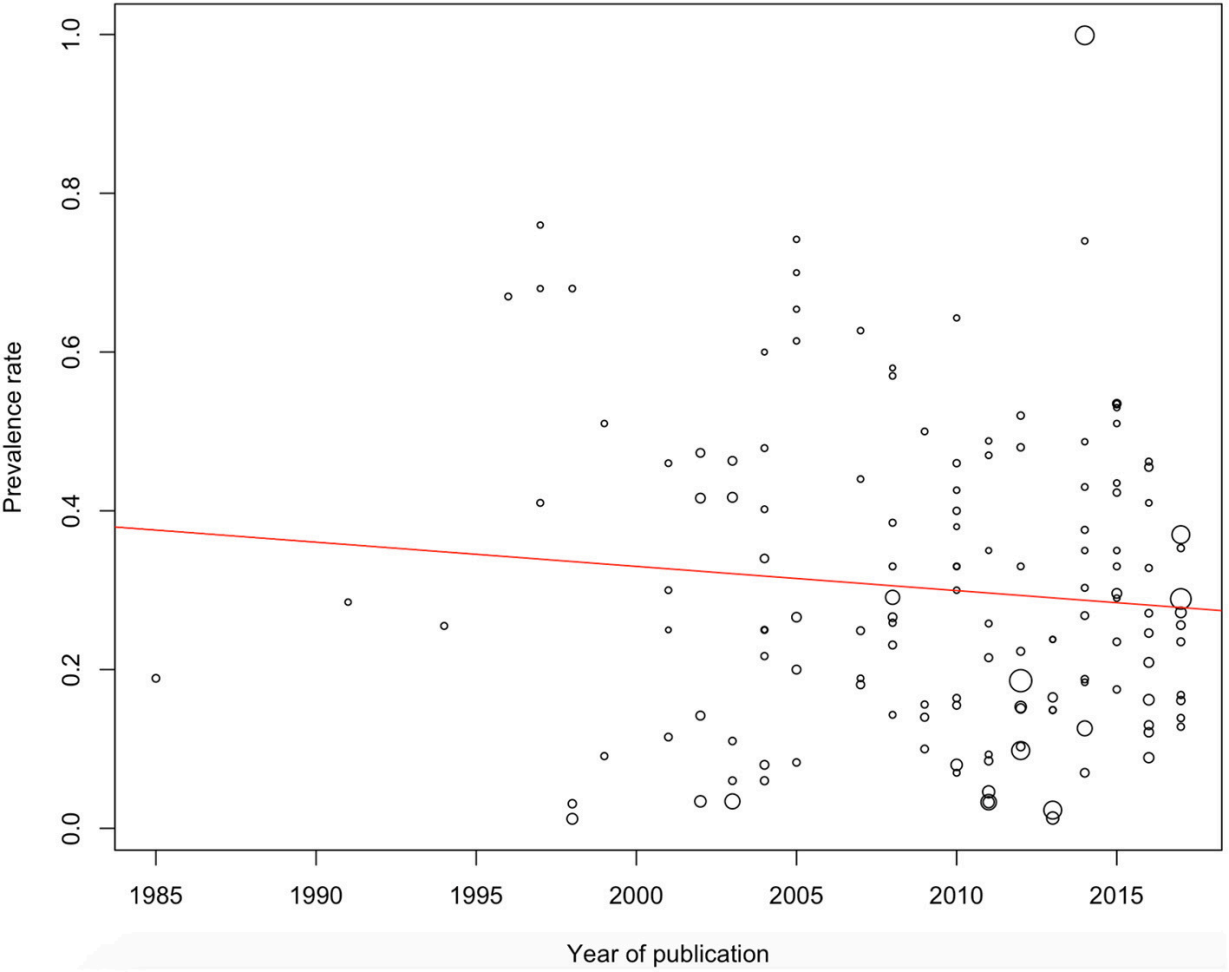
ADHD prevalence estimates according to publication year. The point sizes correspond to the standard error (a larger size indicates a higher error).

The ADHD adolescent/adult meta-analytic prevalence estimate was 26.2% [95% confidence interval (CI): 22.7–29.6]. The childhood ADHD meta-analytic prevalence estimate assessed retrospectively in adolescence/adulthood was 41.1% (95% CI: 34.9–47.2). Data based on clinical interviews (83 study samples) showed an overall prevalence estimate of 26.7% (95% CI: 22.7–30.7). Prevalence estimates for all study samples according to the year of publication are reported in Figure 2.

### Factors related to ADHD prevalence estimate

Results of meta-regressions for all studies (*n* = 142 samples) are reported in Table 1. Only one covariate was significantly associated with heterogeneity of prevalence estimates. Screenings of childhood ADHD were associated with an increased prevalence rate compared to current diagnosis using clinical interviews (i.e., for the univariate model: respectively b = 0.28, which correspond to a prevalence estimate of 28% and b = 0.15, which corresponded to a prevalence estimate of 0.28 + 0.15 = 43%, *p* < 0.001) or screening of adolescent/adult ADHD (univariate and multivariate models: estimate = 0.17, *p* < 0.001, not shown in Table 1). For the diagnosis of adolescent/adult ADHD, there was no difference between clinical interviews and screenings (*p* ≥ 0.574).

When pooling only articles using diagnostic interviews (*n* = 83), the results were almost similar, with no covariate reaching the significance level (Table 2). For models with significant predictors, the VAF remained small (VAF ≤ 10.3%).

**Table 2:** Univariate and multivariate meta-regressions for study samples with clinical interviews (*n* = 83).

	**No. of study samples**	**Univariate models**			**Multivariate model**		
		**Estimate**	***p*-value**	**VAF%**	**Estimate**	***p*-value**	**VAF%**
Intercept	−	–	–	–	**0.20**	**<0.001**	**3.5**
**Region**							
North America (reference)	40	**0.27**	**<0.001**	2.4	–	–	
Asia	7	0.10	0.203		–	–	
Australia	4	−0.15	0.115		–	–	
Europe	32	0.02	0.600		–	–	
South America	0	–	–		–	–	
**Gender**							
Male (reference)	59	**0.27**	**<0.001**	0.0	–	–	
Female	24	−0.01	0.949		0.01	0.896	
**Study population**							
Adults (reference)	28	**0.22**	**<0.001**	2.2	–	–	
Youths	55	0.06	0.133		0.07	0.105	
**Psychiatric diagnosis**							
No (reference)	63	**0.25**	**<0.001**	2.3	–	–	
Yes	20	0.09	0.073		0.09	0.057	
**Serious offenders**							
No (reference)	71	**0.27**	**<0.001**	0.0	–	–	
Yes	12	−0.05	0.361		−0.05	0.433	
**DSM Version**							
DSM-IV (reference)	65	**0.26**	**<0.001**	0.0	–	–	
DSM-III	10	−0.02	0.718		–	–	
DSM-5	3	0.11	0.331		–	–	
**Quality**							
Strong (reference)	54	**0.26**	**<0.001**	0.0	–	–	
Moderate	25	0.04	0.339		–	–	
Weak	4	−0.05	0.676		–	–	

VAF, Variance accounted for (pseudo-R^2^).

*Significant results are in bold*.

## Discussion

### ADHD prevalence rate among people living in detention

This study updated the prevalence rate of ADHD in prison settings (including PLD detained in psychiatric units). We identified 102 studies meeting study criteria (142 study samples) published from 1985 to 2017 with data collected in 28 countries. The pooling of all studies yielded an adolescent/adult ADHD prevalence rate of 26.2%, while the pooling of only those using clinical interviews found a similar rate of 26.7%. This high ADHD prevalence rate corresponds with a five-fold increase in comparison with that of the general population (12–14). These findings are consistent with those of Young et al's earlier meta-analysis (31) and added more evidence for the relationship between ADHD and involvement in the legal system.

By contrast, the retrospective assessment of ADHD in childhood was higher at 41.1%. This suggests a remission rate of 63.8%, although a study with a longitudinal design would be required to definitively confirm this. Nevertheless, the estimation corresponds to data obtained from the general population reporting remission in 40–60% of cases (1).

These results suggest that PLD bear a heavy mental health burden on secure services as around one-third may require treatment for ADHD. PLD with ADHD should be referred to mental health services, not only to confer personal health and well-being, but because treatment may support them in their interface and progress within the criminal justice system (6, 11). Several studies have reported the efficacy and safety of pharmacotherapy for ADHD during adulthood, also in prison (32–35). These studies reported strong treatment effects with positive outcomes (e.g., reduction in the symptomatology of ADHD over time, no drug abuse during the study, increase in psychosocial outcome). Most of the studies included in our meta-analysis did not report ADHD treatment. Therefore, intervention studies to treat ADHD in prison are needed in addition to screening and diagnostic studies.

Furthermore, the meta-analysis of Young et al. (36) reported that PLD with ADHD (compared with those without ADHD) had significantly higher rates of mood disorder in youth institutions and those in adult institutions presented with significantly higher rates of conduct disorder in childhood, anxiety, mood, personality, and substance use disorders. Hence, they are individuals with a higher rate of comorbidity. Even if some specific psychological interventions have been developed for youths and adults with ADHD [e.g., (31, 37, 38)], there is a dearth of data on both pharmacological and psychological intervention for people with ADHD in the criminal justice system and this should be investigated as a priority, given the high prevalence of ADHD in detainees.

### Comparing ADHD assessments

Our study results are influenced by the methods used to ascertain ADHD. Screenings for ADHD in childhood were associated with increased prevalence estimates in comparison with evaluation of adolescent/adult ADHD (using clinical interviews and self-reported screenings). Previous studies already demonstrated that methods have an effect on the prevalence estimate (5, 12). This result was probably due to the fact that some participants were in remission from ADHD (39, 40). Consequently, prevalence rates for childhood and adult ADHD should not be grouped together. Attention should be given to the kind of assessment used to estimate the prevalence rate of ADHD when interpreting the data.

Prevalence estimates pooled from studies using screenings for adolescence/adult ADHD were not significantly different from estimates of studies using clinical interviews. Recent studies showed that self-reported assessments of ADHD in adulthood are reliable [for example, the ADHD self-reported screening scale, ASRS, (41): sensitivity = 91.4%, specificity = 96.0%; the Barkley screen (B-BAARS) (42): sensitivity = 84%, specificity = 82%]. Our results suggest the same conclusions. Using self-reported screening may be a reliable way of assessing adult ADHD, although caution is needed because a complete evaluation by an experienced clinician is required to provide a formal diagnosis. Clinical interviews may also find psychiatric comorbid states.

To our knowledge, no meta-analysis has investigated differences in estimates according to DSM versions, including the DSM-5. Unfortunately, there were only three studies using the DSM-5 version to assess ADHD among PLD. There was no significant difference between the versions of DSM, but there was probably a bias due to a lack of statistical power. Further studies should test whether there is an increase in the prevalence rate of ADHD when the DSM-5 definition is used (4, 21). We recommend that all future studies use the DSM-5 to provide unbiased prevalence rates of ADHD (41).

From a methodological point of view, the quality of the studies did not significantly affect the prevalence estimates of ADHD (presence of exclusion criteria, high non-response rate, or convenient samples, and use of self-reported screenings). Previous studies criticized methodological weaknesses in many prison (22, 43). However, our meta-analysis pooled studies of generally high quality that used reliable and valid ADHD diagnostic approaches as well as robust methods altogether agreeing a high ADHD prevalence estimate.

### ADHD according to participant characteristics

One of our sub-objectives was to identify which characteristics of PLD were associated with ADHD. Among the five characteristics included in our meta-analysis, none was associated with a significant increase in the ADHD prevalence rate.

Although ADHD is highly comorbid with other psychiatric disorders (36, 44), we did not identify a significant increase of ADHD among PLD with a comorbid diagnostic. In incarcerated populations, Young et al. (36) reported that several psychiatric disorders co-occur with an ADHD diagnostic in PLD, including conduct disorder, substance use disorder, mood disorder, depressive disorder, anxiety disorder, and personality disorder. In our study, “PLD having a psychiatric disorder” included a large range of disorders (conduct disorder, substance use disorder, mood disorder, and personality disorder). Our non-significant result might suggest that PLD without a formal diagnosis of comorbid disorders, not detained in psychiatric units or who have not been referred for psychiatric forensic investigation may in fact also have psychiatric comorbidities. This would in turn suggests that PLD are a highly comorbid population as a whole and that attention should be given to ADHD even if no other formal diagnosis exists. Another explanation was that ADHD is comorbid with some specific disorders (e.g., substance use disorders or antisocial personality disorder). Theses specific features have been missed since the comorbidity group included all psychiatric conditions in a general way.

There were no significant differences for gender and age (adults vs. youths). This supported the previous meta-analysis of Young et al. (5) conducted on a prison population and contrasted with findings from the general population (reporting higher prevalence of ADHD amongst males and youths). Regarding gender, a previous study reported that the prevalence rate ranges from 2.1 to 5.4% among males and 1.1 to 3.2% among females, but females were more likely to have persistent ADHD in adulthood (45). However, this narrative review reported that gender differences may be partially due to methodological bias rather than fundamental differences in the expression of ADHD. For example, males may be over-referred and over-diagnosed in comparison with females (45–47). This referral bias is lost with offenders, because female offenders become noticed due to their offending. Another explanation is the 8:1 ratio of males to females living in detention may mean that females benefit from protective measures that keep them out of detention (5). Female offenders are therefore likely to be more serious cases with a high rate of psychiatric disorders, including ADHD, in comparison with male offenders (48, 49).

Regarding population age, studies have reported that full remission of childhood ADHD commonly occurred in adulthood after brain maturation (39, 40). However, we did not find any difference between adolescents (mean age = 16.2) and adults. The higher prevalence rates for childhood prevalence in comparison with adolescence/adulthood prevalence may be due to the remission between childhood and adulthood. Another explanation may be that young offenders with ADHD are diverted out of the criminal justice system and referred early on to psychiatric outpatient clinics or adapted residential homes (5). Further, adults with ADHD may be over-represented in prison settings in comparison with the general population, because ADHD symptoms is associated with an increased risk of offending (5–7).

## Limitations

Only methodological and PLD characteristics that were available across studies, or in most studies, were included. This may have led us to miss some important factors associated with the heterogeneity of study findings. For example, we were unable to extract precisely the type of detention (e.g., pre-trial, post-trial, high-security prison). Most studies did not report ADHD treatment, which may have been helpful in understanding ADHD remission. Second, the validity and reliability of ADHD assessments may have contributed to the heterogeneity of the prevalence estimates. However, our meta-analysis took into account the overall diagnostic approach, even if the specific characteristics of the assessment scales were not included in the model. The heterogeneity of the samples used was also a possible source of variability in the prevalence estimates. This was taken into account by using random study effects. Finally, there were insufficient studies applying the DSM-5 definition of ADHD for meaningful analysis and some regions of the world were under-represented (South America), whereas other were completely missing (e.g., Africa).

## Conclusion

ADHD has been an important research focus in the last 2 decades, with 102 studies published in 28 countries regarding prevalence in prison settings around the world. In light of the high ADHD prevalence among PLD (including PLD with comorbid disorders incarcerated in psychiatric units), a five-fold increase in comparison with the general population, our results reinforce the importance of addressing this critical public health issue by (i) systematically offering ADHD screening and diagnosis to all individuals entering detention (youths, adults, men, women) following the most up-to-date criteria, and (ii) delivering treatment, monitoring, and care for ADHD and other psychiatric comorbidities to patients while they are in prison and after their release. These strategies may benefit PLD, prison staff, and society in general. Further studies should research the needs of this population and investigate the efficacy and effectiveness of treatment (both pharmacological and psychological) for PDL with ADHD are required.

## Author contributions

SB, AF, PH, DD, and HW conceived the study. SB, AF, MG, EF, DG, NTT, and HW collected data. SB drafted the manuscript and performed statistical analyses. All authors excepted SB made substantial contributions for the interpretation of data and revised the manuscript critically for important intellectual content. All authors approved the final version to be published and agreed to be accountable for all aspects of the work related to its accuracy and integrity.

### Conflict of interest statement

The authors declare that the research was conducted in the absence of any commercial or financial relationships that could be construed as a potential conflict of interest. The reviewer DD and handling editor declared their shared affiliation at time of review.
