# Supplementary material for: Prevalence of Attention Deficit Hyperactivity Disorder in Detention Settings: A Systematic Review and Meta-Analysis
Source: Front Psychiatry. 2018 Aug 2;9:331. doi: 10.3389/fpsyt.2018.00331 (PMC6084240; doi:10.3389/fpsyt.2018.00331)
Supplement: Supplementary file 1 [file Data_Sheet_1.docx]

Appendix 1. Criteria for bias assessment

| **Criteria** | |  |
| --- | --- | --- |
| Sampling | |  |
|  | All PLD invited to participate | strong |
|  | Convenient sample | weak |
| Study participation (when all PLD invited to participate) | |  |
|  | ≥ 60% | strong |
|  | < 60% | weak |
| Exclusion criteria: exclude non-native speakers and PLD with disorders | |  |
|  | 0 | strong |
|  | 1 or 2 | weak |
| Outcome measurement | |  |
|  | Clinical interview | strong |
|  | Screening (current or retrospective) | weak |
| **Bias evaluation** | |  |
|  | 0 "weak" | strong |
|  | 1 "weak" | moderate |
|  | ≥ 2 "weak" | weak |

PLD: People living in detention
