# Supplementary material for: Prevalence of Attention Deficit Hyperactivity Disorder in Detention Settings: A Systematic Review and Meta-Analysis
Source: Front Psychiatry. 2018 Aug 2;9:331. doi: 10.3389/fpsyt.2018.00331 (PMC6084240; doi:10.3389/fpsyt.2018.00331)
Supplement: Supplementary file 2 [file Data_Sheet_2.docx]

**Appendix 2 – list of references**

1. Abracen J, Langton CM, Looman J, Gallo A, Ferguson M, Axford M, et al. Mental health diagnoses and recidivism in paroled offenders. International Journal of Offender Therapy and Comparative Criminology. 2014;58(7):765–79.

2. Teplin LA, Abram KM, McClelland GM, Dulcan MK. Comorbid psychiatric disorders in youth in juvenile detention. Archives of General Psychiatry. 2003 Nov;60(11):1097–108.

3. Abramowitz CS, Kosson DS, Seidenberg M. The relationship between childhood Attention Deficit Hyperactivity Disorder and conduct problems and adult psychopathy in male inmates. Personality and Individual Differences. 2004 Mar;36(5):1031–47.

12. Asbjornsen AE, Manger T, Eikeland O-J. Symptoms of ADHD are Related to Education and Work Experience Among Incarcerated Adults. Journal of Prison Education and Reentry. 2015 Jun;2(1):18–30.

13. Asbjornsen AE, Jones LO, Munkvold LH, Obrzut JE, Manger T. An Examination of Shared Variance in Self-Report and Objective Measures of Attention in the Incarcerated Adult Population. Journal of Attention Disorders. 2010 Sep;14(2):182–93.

17. Baglivio MT, Wolff KT, Piquero AR, Greenwald MA, Epps N. Racial/Ethnic Disproportionality in Psychiatric Diagnoses and Treatment in a Sample of Serious Juvenile Offenders. Journal of Youth and Adolescence. 2017 Jul;46(7):1424–51.

18. Baglivio MT, Wolff KT, Piquero AR, DeLisi M, Vaughn MG. Multiple Pathways to Juvenile Recidivism: Examining Parental Drug and Mental Health Problems, and Markers of Neuropsychological Deficits Among Serious Juvenile Offenders. Criminal Justice and Behavior. 2017 Aug;44(8):1009–29.

40. Beck NC, Hammer JH, Robbins S, Tubbesing T, Menditto A, Pardee A. Highly Aggressive Women in a Forensic Psychiatric Hospital. Journal of the American Academy of Psychiatry and the Law. 2017 Mar 1;45(1):17–24.

52. Bielas H, Barra S, Skrivanek C, Aebi M, Steinhausen H-C, Bessler C, et al. The associations of cumulative adverse childhood experiences and irritability with mental disorders in detained male adolescent offenders. Child and Adolescent Psychiatry and Mental Health. 2016 Sep 22;10:34.

54. Bijleveld C, Van Der Geest V, Hendriks J. Bullying and (re)offending: Results from three samples in the Netherlands. Criminal Behaviour and Mental Health. 2011;21(2):145–50.

55. Billstedt E, Anckarsater H, Wallinius M, Hofvander B. Neurodevelopmental disorders in young

85. Byrne MK, Parletta N, Webster DG, Batterham M, Meyer BJ. Adult attention deficit disorder and aggressive behaviour: An exploration of relationships between Brown Attention-Deficit Disorder Scales and the Aggression Questionnaire. Psychiatry, Psychology and Law. 2015;22(3):407–16.

86. Cahill BS, Coolidge FL, Segal DL, Klebe KJ, Marle PD, Overmann KA. Prevalence of ADHD and its subtypes in male and female adult prison inmates. Behavioral Sciences & the Law. 2012;30(2):154–66.

99. Chang H-L, Chen S-H, Huang C. Temperament of juvenile delinquents with history of substance abuse. Chang Gung Med J. 2007 Feb;30(1):47–52.

112. Colins OF, Andershed H. The DSM-5 With Limited Prosocial Emotions Specifier for Conduct Disorder Among Detained Girls. Law and Human Behavior. 2015 Apr;39(2):198–207.

113. Colins O, Vermeiren R, Schuyten G, Broekaert E, Soyez V. Informant agreement in the assessment of disruptive behavior disorders in detained minors in Belgium: A diagnosis-level and symptom-level examination. The Journal of Clinical Psychiatry. 2008;69(1):141–8.

117. Comai S, Bertazzo A, Vachon J, Daigle M, Toupin J, Cote G, et al. Tryptophan via serotonin/kynurenine pathways abnormalities in a large cohort of aggressive inmates: markers for aggression. Progress in Neuro-Psychopharmacology & Biological Psychiatry. 2016 Oct 3;70:8–16.

119. Conner AC, Kissling C, Hodges E, Hünnerkopf R, Clement RM, Dudley E, et al. Neurotrophic factor-related gene polymorphisms and adult attention deficit hyperactivity disorder (ADHD) score in a high-risk male population. American Journal of Medical Genetics Part B: Neuropsychiatric Genetics. 2008;147B(8):1476–80.

129. Cropsey KL, Linker JA, Waite DE. An analysis of racial and sex differences for smoking among adolescents in a juvenile correctional center. Drug Alcohol Depend. 2008 Jan 1;92(1–3):156–63.

135. Curran S, Fitzgerald M. Attention deficit hyperactivity disorder in the prison population. Am J Psychiatry. 1999 Oct;156(10):1664–5.

136. Dåderman AM, Lindgren M, Lidberg L. The prevalence of dyslexia and AD/HD in a sample of forensic psychiatric rapists. Nordic Journal of Psychiatry. 2004 Oct;58(5):371–81.

142. Dalteg A, Levander S. Twelve thousand crimes by 75 boys: a 20-year follow-up study of childhood hyperactivity. Journal of Forensic Psychiatry. 1998 May;9(1):39–57.

143. Dalteg A, Lindgren M, Levander S. Retrospectively rated ADHD is linked to specific personality characteristics and deviant alcohol reactions. Journal of Forensic Psychiatry. 1999 Dec;10(3):623–34.

144. Dalteg A, Zandelin A, Tuninger E, Levander S. Psychosis in adulthood is associated with high rates of ADHD and CD problems during childhood. Nordic Journal of Psychiatry. 2014;68(8):560–6.

157. DeYoung CG, Getchell M, Koposov RA, Yrigollen CM, Haeffel GJ, af Klinteberg B, et al. Variation in the catechol-O-methyltransferase Val(158)Met polymorphism associated with conduct disorder and ADHD symptoms, among adolescent male delinquents. Psychiatric Genetics. 2010 Feb;20(1):20–4.

166. Edvinsson D, Bingefors K, Lindstrom E, Lewander T. ADHD-related symptoms among adults in out-patient psychiatry and female prison inmates as compared with the general population. Ups J Med Sci. 2010 Feb;115(1):30–40.

168. Einarsson E, Sigurdsson JF, Gudjonsson GH, Newton AK, Bragason OO. Screening for attention-deficit hyperactivity disorder and co-morbid mental disorders among prison inmates. Nordic Journal of Psychiatry. 2009;63(5):361–7.

169. Einat T, Einat A. Learning disabilities and delinquency: a study of Israeli prison inmates. Int J Offender Ther Comp Criminol. 2008 Aug;52(4):416–34.

186. Eyestone LL, Howell RJ. An epidemiological study of attention-deficit hyperactivity disorder and major depression in a male prison population. Bull Am Acad Psychiatry Law. 1994;22(2):181–93.

197. Farooq R, Emerson L-M, Keoghan S, Adamou M. Prevalence of adult ADHD in an all-female prison unit. Atten Defic Hyperact Disord. 2016 Jun;8(2):113–9.

216. FOREHAND R, WIERSON M, FRAME C, KEMPTON T, ARMISTEAD L. JUVENILE-DELINQUENCY ENTRY AND PERSISTENCE - DO ATTENTION PROBLEMS CONTRIBUTE TO CONDUCT PROBLEMS. Journal of Behavior Therapy and Experimental Psychiatry. 1991 Dec;22(4):261–4.

227. Gaïffas A, Galéra C, Mandon V, Bouvard MP. Attention‐deficit/hyperactivity disorder in young French male prisoners. Journal of Forensic Sciences. 2014;59(4):1016–9.

242. Ginsberg Y, Hirvikoski T, Lindefors N. Attention Deficit Hyperactivity Disorder (ADHD) among longer-term prison inmates is a prevalent, persistent and disabling disorder. BMC Psychiatry. 2010;10.

250. González RA, Vélez-Pastrana MC, Ruiz Varcárcel JJ, Levin FR, Albizu-García CE. Childhood ADHD symptoms are associated with lifetime and current illicit substance-use disorders and in-site health risk behaviors in a representative sample of Latino prison inmates. Journal of Attention Disorders. 2015;19(4):301–12.

251. Goodwin E, Gudjonsson GH, Sigurdsson JF, Young S. The impact of ADHD symptoms on intelligence test achievement and speed of performance. Personality and Individual Differences. 2011;50(8):1273–7.

252. Gordon JA, Diehl RL, Anderson L. Does ADHD matter? Examining attention deficit and hyperactivity disorder on the likelihood of recidivism among detained youth. Journal of Offender Rehabilitation. 2012;51(8):497–518.

253. Gordon V, Donnelly PD, Williams DJ. Relationship between ADHD symptoms and anti-social behaviour in a sample of older youths in adult Scottish prisons. Personality and Individual Differences. 2014;58:116–21.

263. Grieger L, Hosser D. Attention deficit hyperactivity disorder does not predict criminal recidivism in young adult offenders: Results from a prospective study. International Journal of Law and Psychiatry. 2012;35(1):27–34.

280. Gunter TD, Arndt S, Wenman G, Allen J, Loveless P, Sieleni B, et al. Frequency of mental and addictive disorders among 320 men and women entering the Iowa prison system: Use of the MINI-Plus. Journal of the American Academy of Psychiatry and the Law. 2008;36(1):27–34.

285. Hamzeloo M, Mashhadi A, Fadardi JS. The prevalence of ADHD and comorbid disorders in Iranian adult male prison inmates. Journal of Attention Disorders. 2016;20(7):590–8.

293. Harzke AJ, Baillargeon J, Baillargeon G, Henry J, Olvera RL, Torrealday O, et al. Prevalence of psychiatric disorders in the Texas juvenile correctional system. Journal of Correctional Health Care. 2012;18(2):143–57.

295. Haysom L, Indig D, Moore E, Gaskin C. Intellectual disability in young people in custody in New South Wales, Australia - prevalence and markers. Journal of Intellectual Disability Research. 2014 Nov;58(11):1004–14.

300. Hennessey KA, Stein MD, Rosengard C, Rose JS, Clarke JG. Childhood Attention Deficit Hyperactivity Disorder, Substance Use, and Adult Functioning Among Incarcerated Women. Journal of Attention Disorders. 2010 Nov;14(3):273–80.

337. Karnik NS, Soller MV, Redlich A, Silverman MA, Kraemer HC, Haapanen R, et al. Prevalence Differences of Psychiatric Disorders among Youth after Nine Months or more of Incarceration by Race/Ethnicity and Age. Journal of Health Care for the Poor and Underserved. 2010 Feb;21(1):237–50.

338. Kavanagh L, Rowe D, Hersch J, Barnett KJ, Reznik R. Neurocognitive deficits and psychiatric disorders in a NSW prison population. International Journal of Law and Psychiatry. 2010 Feb;33(1):20–6.

342. Khanna D, Shaw J, Dolan M, Lennox C. Does diagnosis affect the predictive accuracy of risk assessment tools for juvenile offenders: Conduct Disorder and Attention Deficit Hyperactivity Disorder. Journal of Adolescence. 2014 Oct;37(7):1171–9.

343. Kim JI, Kim B, Kim B-N, Hong S-B, Lee DW, Chung J-Y, et al. Prevalence of psychiatric disorders, comorbidity patterns, and repeat offending among male juvenile detainees in South Korea: A cross-sectional study. Child and Adolescent Psychiatry and Mental Health. 2017;11.

353. Konstenius M, Larsson H, Lundholm L, Philips B, van de Glind G, Jayaram-Lindstrom N, et al. An Epidemiological Study of ADHD, Substance Use, and Comorbid Problems in Incarcerated Women in Sweden. Journal of Attention Disorders. 2015 Jan;19(1):44–52.

365. Langevin R, Curnoe S. Are Dangerous Offenders Different From Other Offenders? A Clinical Profile. International Journal of Offender Therapy and Comparative Criminology. 2014 Jul;58(7):780–801.

398. Maniadaki K, Kakouros E. Attention problems and learning disabilities in young offenders in detention in Greece. Psychology. 2011;2(1):53–9.

409. Matsumoto T, Yamaguchi A, Asami T, Kamijo A, Iseki E, Hirayasu Y, et al. Drug preferences in illicit drug abusers with a childhood tendency of attention deficit/hyperactivity disorder: a study using the Wender Utah Rating Scale in a Japanese prison. Psychiatry Clin Neurosci. 2005 Jun;59(3):311–8.

415. McCarthy J, Chaplin E, Underwood L, Forrester A, Hayward H, Sabet J, et al. Characteristics of prisoners with neurodevelopmental disorders and difficulties. Journal of Intellectual Disability Research. 2016;60(3):201–6.

429. Moore E, Sunjic S, Kaye S, Archer V, Indig D. Adult ADHD among NSW prisoners: Prevalence and psychiatric comorbidity. Journal of Attention Disorders. 2016;20(11):958–67.

435. Mundt AP, Alvarado R, Fritsch R, Poblete C, Villagra C, Kastner S, et al. Prevalence rates of mental disorders in Chilean prisons. PLoS ONE. 2013;8(7).

448. Olvera RL, Semrud-Clikeman M, Pliszka SR, O’Donnell L. Neuropsychological deficits in adolescents with conduct disorder and comorbid bipolar disorder: a pilot study. Bipolar Disorders. 2005 Feb;7(1):57–67.

463. Plattner B, The SSL, Kraemer HC, Williams RP, Bauer SM, Kindler J, et al. Suicidality, psychopathology, and gender in incarcerated adolescents in Austria. The Journal of Clinical Psychiatry. 2007;68(10):1593–600.

466. Pondé MP, Freire ACC, Mendonça MSS. The prevalence of mental disorders in prisoners in the city of Salvador, Bahia, Brazil. Journal of Forensic Sciences. 2011;56(3):679–82.

481. Rasmussen K, Almvik R, Levander S. Attention deficit hyperactivity disorder, reading disability, and personality disorders in a prison population. J Am Acad Psychiatry Law. 2001;29(2):186–93.

485. Rayner J, Kelly TP, Graham F. Mental health, personality and cognitive problems in persistent adolescent offenders require long-term solutions: a pilot study. Journal of Forensic Psychiatry & Psychology. 2005 Jun;16(2):248–62.

505. Román‐Ithier JC, González RA, Vélez‐Pastrana MC, González‐Tejera GM, Albizu‐García CE. Attention deficit hyperactivity disorder symptoms, type of offending and recidivism in a prison population: The role of substance dependence. Criminal Behaviour and Mental Health. 2016;27(5);443.456.

507. Rösler M, Retz W, Retz-Junginger P, Hengesch G, Schneider M, Supprian T, et al. Prevalence of attention deficit-/hyperactivity disorder (ADHD) and comorbid disorders in young male prison inmates. European Archives of Psychiatry and Clinical Neuroscience. 2004;254(6):365–71.

508. Rösler M, Retz W, Yaqoobi K, Burg E, Retz-Junginger P. Attention deficit/hyperactivity disorder in female offenders: Prevalence, psychiatric comorbidity and psychosocial implications. European Archives of Psychiatry and Clinical Neuroscience. 2009;259(2):98–105.

529. Schier Doria GM, Antoniuk SA, Assumpcao Junior FB, Fajardo DN, Ehlke MN. Delinquency and association with behavioral disorders and substance abuse. Revista da Associacao Medica Brasileira. 2015 Feb;61(1):51–7.

534. Schubert CA, Mulvey EP, Glasheen C. Influence of Mental Health and Substance Use Problems and Criminogenic Risk on Outcomes in Serious Juvenile Offenders. Journal of the American Academy of Child and Adolescent Psychiatry. 2011 Sep;50(9):925–37.

546. Sen P, Arugnanaseelan J, Connell E, Katona C, Khan AA, Moran P, et al. Mental health morbidity among people subject to immigration detention in the UK:  a feasibility study. Epidemiol Psychiatr Sci. 2017 Jun 22;1–10.

555. Siponmaa L, Kristiansson M, Jonson C, Nydén A, Gillberg C. Juvenile and young adult mentally disordered offenders: The role of child neuropsychiatric disorders. Journal of the American Academy of Psychiatry and the Law. 2001;29(4):420–6.

565. Soderstrom H, Sjodin A-K, Carlstedt A, Forsman A. Adult psychopathic personality with childhood-onset hyperactivity and conduct disorder: A central problem constellation in forensic psychiatry. Psychiatry Research. 2004;121(3):271–80.

571. Stahlberg O, Anckarsater H, Nilsson T. Mental health problems in youths committed to juvenile institutions: prevalences and treatment needs. European Child & Adolescent Psychiatry. 2010 Dec;19(12):893–903.

573. Stewart LA, Wilton G, Sapers J. Offenders with cognitive deficits in a Canadian prison population: Prevalence, profile, and outcomes. International Journal of Law and Psychiatry. 2016;44:7–14.

575. Stokkeland L, Fasmer OB, Waage L, Hansen AL. Attention deficit hyperactivity disorder among inmates in Bergen Prison. Scandinavian Journal of Psychology. 2014;55(4):343–9.

585. Teplin LA, Abram KM, McClelland GM, Dulcan MK, Mericle AA. Psychiatric disorders in youth in juvenile detention. Archives of General Psychiatry. 2002 Dec;59(12):1133–43.

602. Unruh D, Bullis M. Female and Male Juvenile Offenders With Disabilities: Differences in the Barriers to Their Transition to the Community. Behavioral Disorders. 2005;30(2):105–17.

603. Usher AM, Stewart LA, Wilton G. Attention deficit hyperactivity disorder in a Canadian prison population. International Journal of Law and Psychiatry. 2013;36(3–4):311–5.

604. Vahl P, Colins OF, Lodewijks HPB, Lindauer R, Markus MT, Doreleijers TAH, et al. Psychopathic traits and maltreatment: Relations with aggression and mental health problems in detained boys. International Journal of Law and Psychiatry. 2016 Jun;46:129–36.

613. Vegue‐González M, Álvaro‐Brun E, Santiago‐Sáez A, Kanaan‐Kanaan A. Retrospective evaluation of attention deficit hyperactivity disorder with the Wender Utah Rating Scale in a sample of Spanish prison inmates. Journal of Forensic Sciences. 2011;56(6):1556–61.

621. Vitelli R. Prevalence of childhood conduct and attention-deficit hyperactivity disorders in adult maximum-security inmates. International Journal of Offender Therapy and Comparative Criminology. 1996 Dec;40(4):263–71.

622. Vitelli R. Comparison of early and late start models of delinquency in adult offenders. International Journal of Offender Therapy and Comparative Criminology. 1997 Dec;41(4):351–7.

624. Vogler N, Perkinson-Gloor N, Brand S, Grob A, Lemola S. Sleep, aggression, and psychosocial adjustment in male prisoners. Swiss Journal of Psychology. 2014;73(3):167–76.

627. Vreugdenhil C, Doreleijers TAH, Vermeiren R, Wouters L, Van den Brink W. Psychiatric disorders in a representative sample of incarcerated boys in the Netherlands. Journal of the American Academy of Child and Adolescent Psychiatry. 2004 Jan;43(1):97–104.

649. Young S, González RA, Mutch L, Mallet-Lambert I, O’Rourke L, Hickey N, et al. Diagnostic accuracy of a brief screening tool for attention deficit hyperactivity disorder in UK prison inmates. Psychological Medicine. 2016;46(7):1449–58.

650. Young S, Gudjonsson G, Ball S, Lam J. Attention Deficit Hyperactivity Disorder (ADHD) in personality disordered offenders and the association with disruptive behavioural problems. Journal of Forensic Psychiatry & Psychology. 2003 Dec;14(3):491–505.

663. Young S, Gudjonsson GH, Wells J, Asherson P, Theobald D, Oliver B, et al. Attention deficit hyperactivity disorder and critical incidents in a Scottish prison population. Personality and Individual Differences. 2009;46(3):265–9.

664. Young S, Gudjonsson G, Misch P, Collins P, Carter P, Redfern J, et al. Prevalence of ADHD symptoms among youth in a secure facility: the consistency and accuracy of self- and informant-report ratings. Journal of Forensic Psychiatry & Psychology. 2010;21(2):238–46.

665. Young S, Gudjonsson G, O’Rourke L, Woodhouse E, Ashwood K, Murphy D, et al. Attention-deficit-hyperactivity disorder and associated functional impairments in mentally disordered offenders. Psychiatry Res. 2015 Dec 15;230(2):387–93.

682. Anckarsäter H, Nilsson T, Ståhlberg O, Gustafson M, Saury JM, Råstam M, Gillberg C. Prevalences and configurations of mental disorders among institutionalized adolescents. Developmental neurorehabilitation. 2007;10(1):57-65.

685. Langevin R. A study of the psychosexual characteristics of sex killers: can we identify them before it is too late? International Journal of Offender Therapy and Comparative Criminology. 2003;47(4):366-382.

687. Kaplan SG, Cornell DG. Psychopathy and ADHD in adolescent male offenders. Youth Violence and Juvenile Justice. 2004;2(2):148-160.

691. Timmons-Mitchell J, Brown C, Schulz SC, Webster SE, Underwood LA, Semple WE. Comparing the mental health needs of female and male incarcerated juvenile delinquents. Behavioral Sciences & the Law. 1997:15(2):195-202.

692. Wasserman GA, McReynolds LS, Lucas CP, Fisher P, Santos L. The voice DISC-IV with incarcerated male youths: prevalence of disorder. Journal of the American Academy of Child & Adolescent Psychiatry. 2002;41(3):314-321.

693. Gosden NP, Kramp P, Gabrielsen G, Sestoft D. Prevalence of mental disorders among 15–17‐year‐old male adolescent remand prisoners in Denmark. Acta Psychiatrica Scandinavica. 2003;107(2):102-110.

694. Ruchkin VV, Schwab-Stone M, Koposov R, Vermeiren R, Steiner H. Violence exposure, posttraumatic stress, and personality in juvenile delinquents. Journal of the American Academy of Child & Adolescent Psychiatry. 2002;41(3):322-329.

695. Duclos CW, Beals J, Novins DK, Martin C, Jewett CS, Manson SM. Prevalence of common psychiatric disorders among American Indian adolescent detainees. Journal of the American Academy of Child & Adolescent Psychiatry. 1998;37(8):866-873.

697. Lederman CS, Dakof GA, Larrea MA, Li H. Characteristics of adolescent females in juvenile detention. International journal of law and psychiatry. 2004;27(4):321-337.

698. Dixon A, Howie P, Starling J. Psychopathology in female juvenile offenders. Journal of Child Psychology and Psychiatry. 2004;45(6):1150-1158.

699. Hollander HE, Turner FD. Characteristics of incarcerated delinquents: Relationship between development disorders, environmental and family factors, and patterns of offense and recidivism. Journal of the American Academy of Child Psychiatry. 1985;24(2):221-226.

700. Gaete J, Labbé N, Villar P, Allende C, Araya R, Valenzuela E. Mental health and associated factors among young offenders in Chile: a cross‐sectional study. *Criminal behaviour and mental health*. 2017.

702. Ghanizadeh A, Nouri SZ, Nabi SS. Psychiatric problems and suicidal behaviour in incarcerated adolescents in the Islamic Republic of Iran. Eastern Mediterranean health journal. 2012;18(4):311.

703. Giotakos O, Markianos M, Vaidakis N. Aggression, impulsivity, and plasma sex hormone levels in a group of rapists, in relation to their history of childhood attention-deficit/hyperactivity disorder symptoms. The Journal of Forensic Psychiatry & Psychology. 2005;16(2):423-433.

705. Bastiaens L, Galus J, Goodlin M. The 12 item WHODAS as primary self report outcome measure in a correctional community treatment center for dually diagnosed patients. Psychiatric Quarterly. 2015;86(2):219-224.

706. Zhou J, Chen C, Wang X, Cai W, Zhang S, Qiu C, Wang H Lu Y, Fazel, S. Psychiatric disorders in adolescent boys in detention: a preliminary prevalence and case–control study in two Chinese provinces. Journal of Forensic Psychiatry & Psychology. 2012;23(5-6):664-675.

730. Machado A, Rafaela D, Silva T, Veigas T, Cerejeira J. (2017). ADHD Among Offenders: Prevalence and Relationship With Psychopathic Traits. *Journal of Attention Disorders*. 2017;1087054717744880.
